# Effect of Ultrasonic Vibration on Adhesive Bonding of CFRP/Al Alloy Joints Grafted with Silane Coupling Agent

**DOI:** 10.3390/polym12040947

**Published:** 2020-04-19

**Authors:** Min Wu, Xuetong Tong, Hui Wang, Lin Hua, Yizhe Chen

**Affiliations:** 1 Hubei Key Laboratory of Advanced Technology for Automotive Components, Wuhan University of Technology, Wuhan 430070, China; wuminmin@whut.edu.cn; 2 Hubei Collaborative Innovation Center for Automotive Components Technology, Wuhan 430070, China; txt0124@whut.edu.cn; 3 Hubei Research Center for New Energy & Intelligent Connected Vehicle, Wuhan University of Technology, Wuhan 430070, China; yzchen@whut.edu.cn

**Keywords:** ultrasonic vibration, adhesive bonding, strengthen, graft, mechanism

## Abstract

Adhesive bonding is widely used in the joining of metals and carbon fiber-reinforced plastics (CFRPs). Ultrasonic vibration was used to improve adhesive bonding of CFRP/Al alloy joints grafted with silane coupling agent, and the effect of the ultrasound on the bonding was studied. The surface of Al alloy was treated with a silane coupling agent, and then the ultrasonic vibration was applied on the adherend during the adhesive bonding process. The shear strength was tested, and the mechanism was analyzed by scanning electron microscope (SEM) and Fourier transform infrared spectrometer (FTIR). It is found that the ultrasonic assisting can further promote the bonding of the Al alloy and the adhesive. For the test joins, the shear strength was increased by 267.50% using the silanization treatment plus the ultrasonic assisting. The ultrasonic assisting promoted the grafted epoxy group to react with the adhesive more sufficiently at the Al/adhesive interface by causing micro-mixing and intensified molecule collision, and thus more chemical bond was formed. Under the ultrasonic action, the interface and the adhesive layer became tighter owing to the impact contact at the interface and the oscillating flow in the adhesive layer. The ultrasonic vibration assisting increased the bonding strength by promoting the chemical bond and improving physical morphology.

## 1. Introduction

Development of the aerospace industry and automobile industry, requiring light weight, high reliability, and good dimensional stability, promotes the application of carbon fiber-reinforced plastics (CFRPs) and light metal materials, such as Al alloy [1,2,3,4]. Joining of CFRP and Al alloy, encountered commonly, has become a critical issue. Compared with conventional mechanical joining approaches, such as bolting and riveting, adhesive bonding stands out for its small stress concentration and light weight. Bonding stress of an adhesive joint can be evenly distributed throughout the bonding area, while the traditional joining approaches easily damage the continuity of load-carrying carbon fibers by puncturing [5,6,7].

Some scholars have studied adhesive bonding. Pantelakis and Tserpes [8] discussed the development and challenge of the adhesive bonding technology for composite materials in aircraft structure and proposed a numerical design method for bonding composite materials to Al alloy. Wang et al. [9] studied the parameters about bonding area, adhesive, surface treatment, and adherend shape to improve the strength. Jeevi et al. [10] discussed parameters influencing the performance of the bonding joints, such as humidity, temperature, and bonding method.

Although adhesive bonding could meet the requirement of lightweight, the bonding strength is still a problem. Surface pretreatment is always needed to avoid the interface failure. Zhang et al. [11] significantly improved the shear strength of the Al alloy/CFRP joint by anodizing pretreatment, which produced a nanoscale porous oxide layer on the surface of the Al alloy A6061. The oxide layer facilitated mechanical anchoring at the interface. Xu et al. [12] studied the permeation behavior of the epoxy resin on the Al alloy sheets, which were pretreated by the phosphoric acid anodic oxidation (PAA) process, in order to improve the bonding property. Chen et al. [13] found that the interfacial bonding strength was significantly increased by polishing and anodic oxidation owing to the generated porous microstructure on the surface. The maximum tensile strength reached 10.05 MPa. 

Silanization treatment is another surface pretreatment method to improve the shear adhesion strength, which can roughen the surface of Al alloy and introduce bonding reaction at the interface. Xu et al. [14] modified the adhesion property of the cerium conversion coating on the Al foil with the silane coupling agent KH570. This showed that the silane coupling agent could effectively improve the adhesion strength between the Al foil and the maleic anhydride grafted polypropylene (PP-*g*-MAH) film owing to the formation of the Si–O–Al bond. Pantoja et al. [15] studied the effect of pH value of silane solution on the bonding performance of electrogalvanized steel. The result showed that the pH value of 4 had a better effect on the bonding performance than pH 6. Picard et al. [16] discussed chemical adhesion of silicone elastomers on primed metal surfaces, and found that the typical primer formulations for the adhesion of the different silicone elastomers on metals could achieve the desired adhesive performance owing to the specific chemistries at the metal surfaces. Pan et al. [17] enhanced the interfacial strength between the AA5083 alloy and the cryogenic adhesive via anodic oxidation and silanization. A chemical bond (Si–O–Al) was formed between the aluminum alloy and the silane film. Lee et al. [18] investigated the adhesion between the Al foil and the cast polypropylene film with maleic anhydride grafted ethylene-propylene-diene monomer (MAH-*g*-EPDM) rubber-based adhesives. They found that the adhesion was improved by imidization between the amine group and the maleic anhydride group if the surface of Al foil was treated with 3-aminopropyl triethoxysilane. Yu and Wan [19] repaired damaged Al alloy plate by glass fiber reinforced polymer (GFRP) bonding. The surface of the Al sheet was treated with the silane coupling agents KH550 and KH560 of different concentrations. The result showed that the repaired samples had a high initial strength, and the heat-resistant properties were also improved.

Grafted silane coupling agent, connecting the inorganic aluminum alloy and organic adhesive, improved the bonding strength of aluminum alloy and adhesive. However, the strength is still not ideal. Good interface contact might not always be maintained. Furthermore, owing to poor transfer of material at the interface, the reaction was always incomplete to form a sufficient chemical bond between the grafted surface and the adhesive. Those limited the improvement of the strength.

Nowadays, ultrasound technology has been widely used in studies and industries. Zhao et al. [20,21] firstly proposed an in situ characterization of microcellular injection molding using ultrasound. Zhang et al. [22] developed a non-destructive method for measuring cavity pressure by evaluating stress on the tie bars of the injection molding machine using ultrasonic technology. Sharma and Luzinov [23] studied the thermal and non-thermal effects of ultrasonic curing and confirmed the forced propagation of epoxy oligomers by ultrasonic vibration. Holtmannspotter et al. [24] presented an approach to improve interface formation prior to the bonding process and to ensure quality of the adhesive bonding by using acoustic cavitation in the liquid adhesive. Barua and Saha [25] utilized ultrasound assisted atomization process for depositing carbon nanotubes (CNTs) on the surface of carbon fiber (CF) cloth, and achieved homogeneous distribution of CNTs, which resulted in increased surface roughness and mechanical interlocking between fibers and matrix. Our previous study proved that ultrasonic vibration can improve wettability and promote chemical reaction owing to its high energy [26,27].

Ultrasonic vibration has functions of interface-contact improvement, mass-transfer enhancement, and chemical reaction promotion, which could be used to deal with problems of bad interface contact and incomplete interface reaction encountered in adhesive bonding of grafted joints. With the problems effectively solved, the bonding strength can be further improved. However, there is no report about the effect of ultrasonic vibration on adhesive bonding of grafted joints, and its mechanism is not clear. In order to improve the bonding strength, it is beneficial to study the effect of ultrasonic vibration on grafted joints.

In this study, ultrasonic vibration was applied to improve adhesive bonding of CFRP/Al alloy joints grafted with a silane coupling agent. The effect of the ultrasound on the bonding was studied. The surface of Al alloy was first treated with a silane coupling agent, and then the ultrasonic vibration was applied on the adherend during the adhesive bonding process. The shear strength was tested, and the mechanism was analyzed in terms of microstructure and chemistry.

This paper is organized as follows. Section 2 describes the ultrasonic assisting method, and gives the experimental scheme. Section 3 gives the results and discussion. Finally, Section 4 summarizes our conclusions.

## 2. Materials and Methods 

### 2.1. Materials

The adherends used in the study were 7075 Al alloy sheets (Shenzhen Hongwang mould Co., Ltd., Shenzhen, China) and CFRP laminates (Wuxi RSN New Material Technology Co., Ltd., Wuxi, China). The reinforcement of the CFRP was 3K-T700 carbon fiber, and the matrix was the bisphenol A-epichlorohydrin epoxy resin. The CFRP laminates were formed by the compression molding technology of prepregs. In one prepreg, carbon fibers were woven as twill fabric with fibers arranged at −45° and 45°. Eighteen plies of the prepregs were compressed in a plate mold at 140 °C for 1 h, and the pressure was 10 MPa. The laminate was then kept in the mold at 90 °C for 8 h to cure it completely. After the curing process, the thickness of the laminate was 2.5 mm, and the tolerance of the thickness measured 0.05 mm. The tensile strength of the laminate was 700 MPa, the elastic modulus was 60 GPa, and the interlaminar shear strength was 55 MPa. The composition of the used 7075 Al alloy is shown in Table 1. The yield strength of the alloy is 503 MPa, the elongation is 11%, the ultimate tensile strength is 572 MPa, and the elastic modulus is 71 GPa.

**Figure 1:**
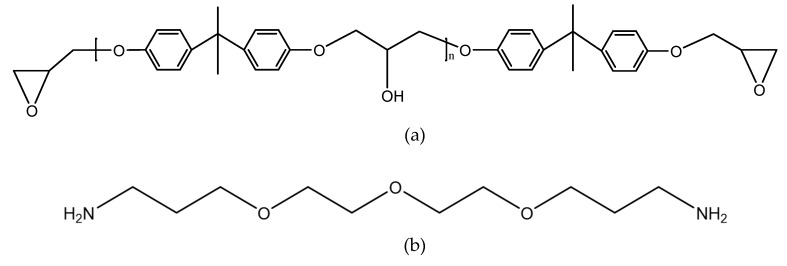

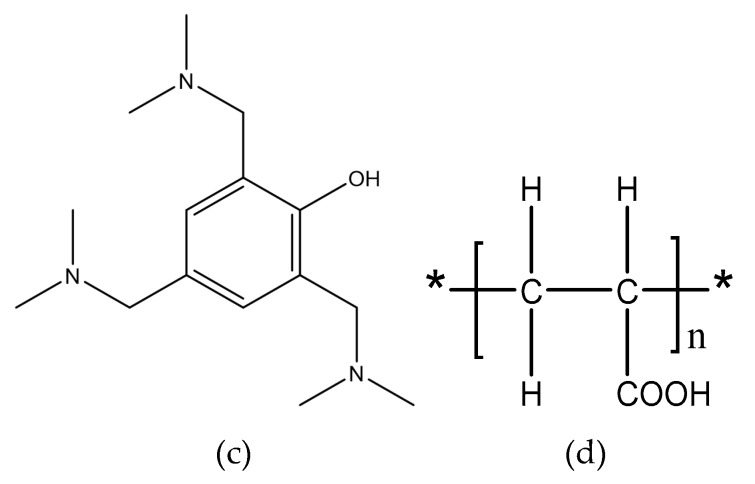
Structural formulas of (**a**) bisphenol A epoxy resin, (**b**) diethylene glycol bis (3-aminopropyl) ether, (**c**) 2,4,6-tris (dimethylaminomethyl) phenol, and (**d**) polyacrylic acid.

The joints were bonded by the two-component adhesive DP460 provided by 3M corporation. The component A of the adhesive mainly contains bisphenol A epoxy resin, while the component B mainly contains diethylene glycol bis (3-aminopropyl) ether, 2,4,6-tris (dimethylaminomethyl) phenol, and polyacrylic acid. The mix ratio of component A and component B is 2:1 by volume. At room temperature, the viscosity of the adhesive is 4.5 Pas, the worklife is 60 min, and the curing time is 4 h. The shear strength is 31 MPa according to the standard ASTM D 1002-72, the T-peel strength is 1071.5 Kg/m according to ASTM D 1876-61T, and the Bell Peel strength is 1375 Kg/m according to ASTM D 3167. The shore D hardness of the cured adhesive is 75–80 HD, and the coefficient of thermal expansion is 59 × 10^−6^/K. The structural formulas are shown in Figure 1, where the functional groups, reacting in the bonding process, are given.

**Figure 2:**
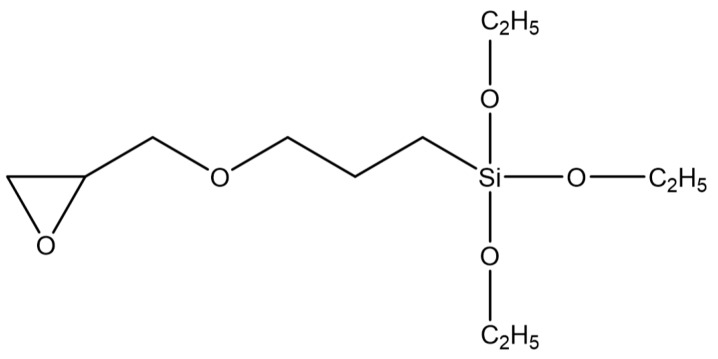
Structural formula of KH560.

The used silane coupling agent was KH560 (Changzhou Runxiang Chemical Co., Ltd., Changzhou, China), and its chemical formula is shown in Figure 2. Other reagents used in the experiment were of analytical grade.

### 2.2. Ultrasonic Vibration Assisting Process

**Figure 3:**
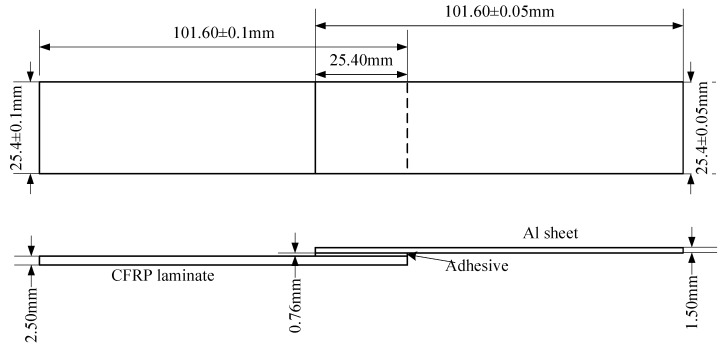
Size of the joint sample. CFRP, carbon fiber-reinforced plastic.

**Figure 4:**
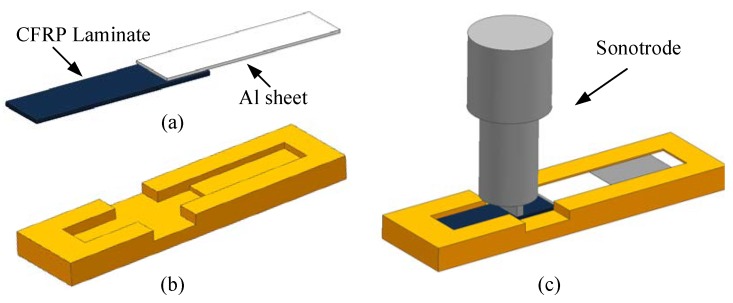
(**a**) The joint, (**b**) the fixture, and (**c**) the schematic diagram of clamping.

According to the standard ASTM D5868-01, the size of the CFRP laminate specimen was 101.6 × 25.4 × 2.5 mm, that of the Al alloy sheet specimen was 101.6 × 25.4 × 1.5 mm, and the size of the bonding layer was 25.4 × 25.4 × 0.76 mm, as shown in Figure 3. Cutting was finished by the suppliers. The CFRP laminates were cut by a NAIK^®^ TC-6060XB computer numerical control (CNC) machine (Shenzhen Tiancheng Xin Li CNC Equipment Co.,Ltd, Shenzhen, China). The used milling cutter was made of tungsten carbide, and its diameter was 1.8 mm. The spindle speed was 2400 r/min, and the feed speed was 400 mm/min. The dimensional accuracy obtained was 0.1 mm. The Al alloy sheets were cut by the MPS-3015D fiber laser cutting machine manufactured by Han’s Laser Technology Co. Ltd. (Shenzhen, China). The laser power used was 500 W, the air pressure was 0.8 MPa, and the cutting speed was 1.5 m/min. The dimensional accuracy obtained was 0.05 mm. To guarantee the required joint size, a fixture, made of 7075 Al alloy, was prepared as shown in Figure 4. In the fixture, the lower cavity was for the CFRP laminate, while the upper was for the Al alloy sheet. This design facilitated the application of the ultrasonic vibration on the CFRP laminate. The height difference of the two cavities was 3.26 mm, the thickness of the CFRP laminate was 2.5 mm, and thus the thickness of 0.76 mm for the adhesive layer was ensured. The structure of the fixture determined the height difference between the CFRP laminate and the Al sheet, and thus determined the thickness of the adhesive layer. The fixture restricted movement and rotation of the adherends. During the experiment, the sonotrode was pressed on the CFRP laminate. The Al sheet was also pressed by a mechanical clamper (not presented), and the pressing position was marked by the gray area in Figure 4c. Therefore, the adherends were clamped by the fixture, the sonotrode, and the mechanical clamper. The manufacturing tolerance of the Al alloy fixture was 0.01 mm. With the fixture, the tolerance of the adhesive joint was 0.06 mm in thickness, 0.16 mm in length, and 0.11 mm in width, considering the adherend tolerance. The tolerance satisfied the requirement of the standard.

**Figure 5:**
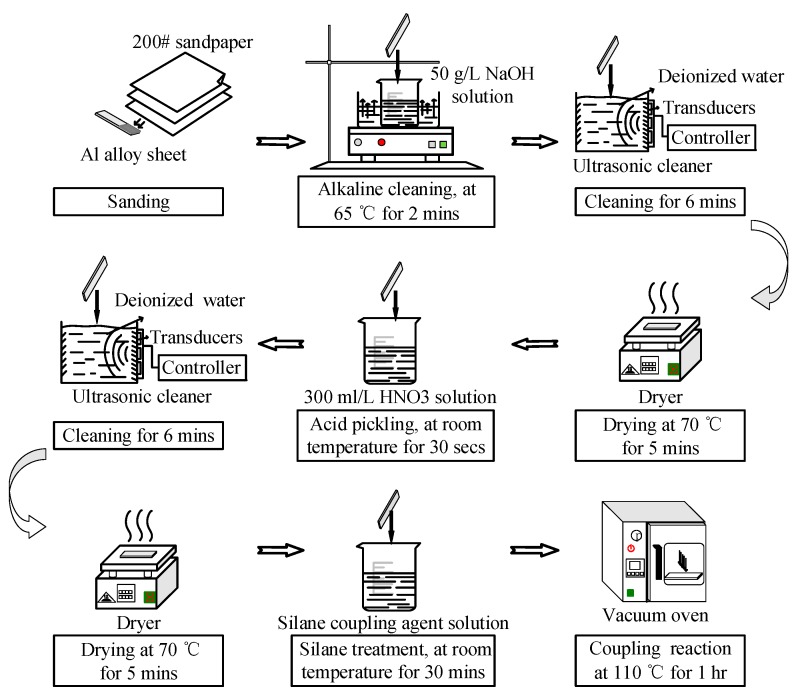
Pretreatment of the Al alloy specimens.

The surface pretreatment of the bonding surface of the Al alloy samples is shown in Figure 5. First, the sanded sample was immersed in 50 g/L NaOH solution (Tianjin Beilian Fine Chemicals Development Co., Ltd., Tianjin, China) at 65 °C for 2 min. Then, the sample was cleaned in an ultrasonic cleaner (Skymen Cleaning Equipment Shenzhen Co. Ltd., Shenzhen, China) with deionized water (Hongwei water treatment equipment Co., Ltd, Guangzhou, China) for 6 min, and dried at 70 °C for 5 min. The power of ultrasonic cleaning was 80 W, and the frequency was 40 kHz. The ultrasonic cleaning was at room temperature, and the water was 3 L (240 × 140 × 100 mm^3^). After the alkaline treatment, the sample was immersed in 300 mL/L HNO_3_ solution (China Pingmei Shenma Group Kaifeng Dongda Chemical Co., Ltd., Kaifeng, China) for 30 s at room temperature. Next, it was cleaned in an ultrasonic cleaner with deionized water for 6 min, and dried at 70 °C for 5 min. Finally, the sample was immersed in silane coupling agent solution for 30 min at room temperature, and kept in a vacuum oven at 110 °C for 1 h to finish the coupling reaction. The silane coupling agent solution was a mixture of methanol (Fangzheng Reagent Factory, Tianjin, China), deionized water, and KH560 with the volume ratio of 96:3:1. The pH value was then adjusted to 5 with acetic acid.

The bonding surface of each CFRP laminate was sanded with 100-mesh sandpaper (3M) in the same direction, and then the surface was cleaned in an ultrasonic cleaner with deionized water for 6 min and with acetone for 6 min sequentially. The power of ultrasonic cleaning was 80 W, and the frequency was 40 kHz. The ultrasonic cleaning was at room temperature, and the cleaning solvent was 3 L (240 × 140 × 100 mm^3^). Finally, the laminate was dried by air naturally for 3 min.

**Figure 6:**
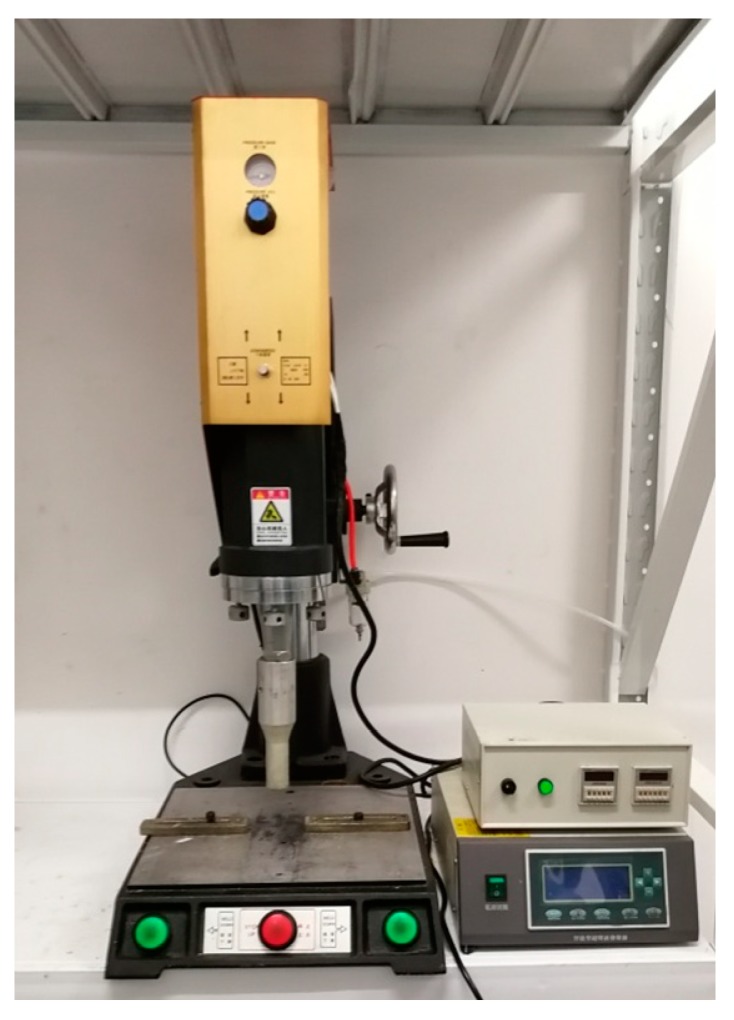
Ultrasonic experiment platform.

The DP460 adhesive was used to join the two adherends, and the ultrasonic vibration was applied during the adhesive bonding process. The ultrasonic equipment (Maxwide Ultrasonic Co., Ltd., Shanghai, China) is shown in Figure 6. The ultrasonic transducer converted high-frequency electrical signals into ultrasonic vibration. The booster, a longitudinal cone type, amplified the mechanical vibration and transmitted it to the sonotrode. The sonotrode, made of 7075 Al alloy, was connected with the lower end of the booster by thread connection. The sonotrode transmitted the vibration to the joint. The CFRP laminate was placed in the fixture, which is shown in Figure 4. The adhesive was evenly dispensed on the bonding area of the CFRP laminate by an EPX™ Plus II manual applicator, manufactured by 3M corporation. The DP460 adhesive was supplied in a dual syringe plastic duo-pak cartridge, and the cartridge was 50 mL. The components A and B were stored in the two syringes, respectively, with a volume ratio of 2:1. If the applicator simultaneously moved the pistons of the two syringes at the same speed, the components were expelled with the desired ratio of 2:1. By a mixing nozzle, the qualified adhesive was obtained. In dispensing of the adhesive, the duo-pak cartridge was first inserted into the applicator, and the plunger was pressed into the cartridge with the trigger of the applicator. Next, the cartridge cap was removed, and a small amount of adhesive was expelled to ensure both sides of the duo-pak cartridge were flowing evenly and freely. Then, a “3.2–16 s” static mixing nozzle was attached to the duo-pak cartridge for dispensing. Finally, the adhesive was dispensed manually on the bonding area according to the space volume of the bonding gap. The dispensing was along a series of lines parallel with the long edge of the joint. Owing to the adjustable flow rate and dispensing speed, the dispensing system can be used to spread the adhesive evenly on the bonding area. After the dispensing was finished, the Al alloy sheet was put inside the fixture to achieve the bond. The fixture with the joint assembly in it was then placed under the sonotrode. The sonotrode was pressed on the CFRP laminate surface to exert the vibration to the joint. The distance was 15 mm from the sonotrode to the bonding area. The vibration was applied for eight cycles. In each cycle, a pause of 1 s was set after the successive vibration of 2 s. The amplitude was 60% of its full potential, the vibration frequency was 15.0 kHz, the current was 2.1 A, and the power was 650 W. After the ultrasonic vibration assisting process, the joint was kept at 30 °C for 5 h to cure the adhesive completely according to the information of the adhesive provided in Section 2.1.

According to the surface treatment of Al alloy and joining process, three different experiments, labelled as Group 1, Group 2, and Group 3, were designed as shown in Table 2. In each group, four samples were prepared, and their labels were also presented in Table 2. The total amount of the samples was 12. Group 1 was a reference experiment, in which the silanization treatment and the ultrasonic vibration were not applied. The treatment parameters were the same as those used in Figure 5. In Group 2, the silanization treatment was applied. The silanization treatment and the ultrasonic vibration were both applied in Group 3.

### 2.3. Characterization

#### 2.3.1. Tensile Test

The microcomputer-controlled electronic universal testing machine of SANS^®^ CMT5202 produced by MTS Systems Co. Ltd. (Shenzhen, China), was used to test the shear strength of each joint. According to the ASTM D5868-01 standard, the test was conducted at room temperature, and the tensile speed was 13 mm/min. Three samples of each group were tested, and the average value was calculated.

#### 2.3.2. Scanning Electron Microscope (SEM) and Energy Dispersive X-Ray Spectroscopy (EDS)

**Figure 7:**
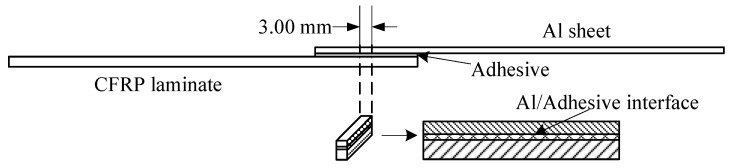
Sample for SEM and EDS test.

The sample for the test is shown in Figure 7. In the middle of a joint, a slice of 3 mm was cut by high pressure waterjet. The cutting surface was sanded with sandpaper of 200#, 400#, 600#, 800#, and 1000#, sequentially, and then polished with flannelette. A layer of 0.1 mm thickness was removed in the process. The Zeiss Ultra Plus Field Emission Scanning Electron Microscope (Carl Zeiss Microscopy GmbH, Jenaer, Germany), equipped with X-Max 50 X-ray spectrometer, was used to observe interface morphology with acceleration voltage of HT = 15.00 kV. Morphology of the interface between the Al sheet and the adhesion was observed, and the element content on the surface of the Al sheet was tested before and after the silanization treatment.

#### 2.3.3. Fourier Transform Infrared Spectroscopy

The change of functional groups at the bonding interface was observed using the Nexus^®^ Smart Fourier Transform Infrared Spectrometer (FTIR) produced by Thermo Electron corporation. The powder was achieved by scraping at the interface, and was then used to prepare KBr pellet to perform the infrared scanning test. The measurements were recorded between 4000 cm^−1^ and 400 cm^−1^ with a resolution of 4 cm^−1^.

## 3. Results and Discussion

### 3.1. Shear Strength and Failure Mode Analysis

The shear strength was measured for three samples of each group, that is, Sample 1, 2, and 3 of Group 1; Sample 5, 6, and 7 of Group 2; and Sample 9, 10, and 11 of Group 3, and the result is shown in Table 3. Sample 4, 8, and 12, which were not subject to the tensile test, were used for the other tests. 

According to the mean value, the shear strength was improved by 215.55%, compared with Group 1, using the silanization treatment, and the strength was improved by 267.50% using the silanization treatment plus the ultrasonic vibration assisting process. In our previous study [27], ultrasonic vibration was used to strengthen the adhesive bonding, but the silanization treatment was not applied. The average shear strength was 10.4 MPa, which was lower than that of Group 3 of this study. It can be confirmed that the strength of the joints prepared by the silanization treatment plus the ultrasonic vibration assisting was obviously higher than those prepared by the two treatments separately.

**Figure 8:**
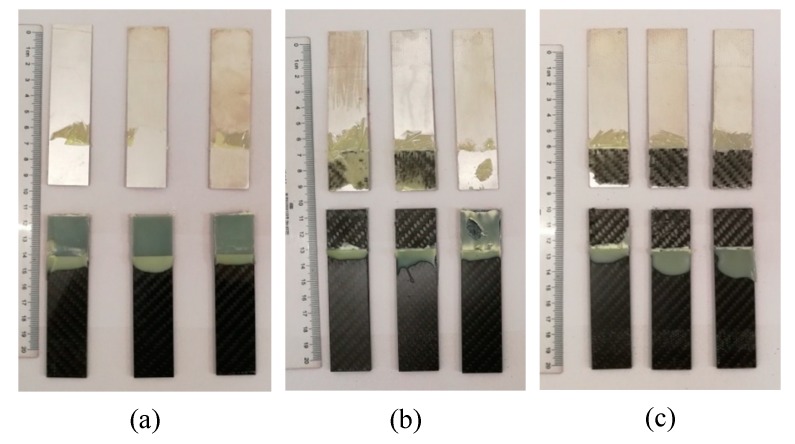
Damaged adhesive layer by tensile test of the samples in (**a**) Group 1, (**b**) Group 2, and (**c**) Group 3.

The damaged adhesive layer is shown in Figure 8. During the tensile test, no obvious damage of the adherends was observed. The maximum shear strength of the joints was 25.46 MPa, which was much lower than the yield strength of the Al alloy (503 MPa, provided in Section 2.1), so no plastic deformation was exhibited for the Al adherends. In addition, the shear strength was lower than the tensile strength (700 MPa) and the interlaminar shear strength (55 MPa) of the CFRP laminates (also provided in Section 2.1), and thus the CFRP adherends were not distinctly damaged. According to the standard ASTM D5573-99, for the samples in Group 1, the separation appeared to be at the Al/adhesive interface, and thereby the failure mode was the adhesive failure (interfacial failure). The shear strength of Group 1 was much lower than the strength of the adhesive (31 MPa, provided in Section 2.1), and thus the adhesive layer was not damaged in the tension process. Because the matrix of the CFRP adherends was similar with the adhesive, the bond between them was much better than that between the Al adherends and the adhesive. The Al alloy was quite different from the adhesive, so the bond between them was very low without the silanization treatment. In the tension test, the Al/adhesive interface was damaged. The shear strength of the samples was increased in Group 2 and Group 3. In Group 2, the right sample exhibited a mixed failure mode of the adhesive failure and the cohesive failure, and the other ones showed a mixed failure mode of the light-fiber-tear failure, the cohesive failure, and the adhesive failure, indicating that the silanization treatment can promote the bonding of the Al alloy and the adhesive. However, the bonding was not ideal. Adhesive failure occurred locally at the Al/adhesive interface. Good interface contact was not fully ensured. Furthermore, owing to the poor transfer of material at the interface, reaction was always incomplete to form a sufficient chemical bond between the grafted surface and the adhesive. Those limited the improvement of the strength. In Group 3, failure mainly occurred within the substrate of CFRP laminates, near the surface. A thin layer of the CFRP resin matrix was visible on the adhesive, with few fibers transferred from the substrate to the adhesive. This failure was typically the light-fiber-tear failure. In this group, the bond between Al adherends and the adhesive was further improved. The strength was larger than the measured shear strength, so the Al/adhesive interface was not damaged in the test. The shear strength was still lower than the strength of the adhesive, but it was certainly larger than the strength of the surface layer of the CFRP adherends. Therefore, the light-fiber-tear failure occurred. From the failure modes, the shear strength of Al alloy and adhesive was low without the silanization treatment. After the treatment, the adherend surface was grafted with the silane coupling agent, which strengthened the bonding between the Al alloy sheet and adhesive. The ultrasonic vibration assisting can further promote the bonding.

### 3.2. Surface Composition 

**Figure 9:**
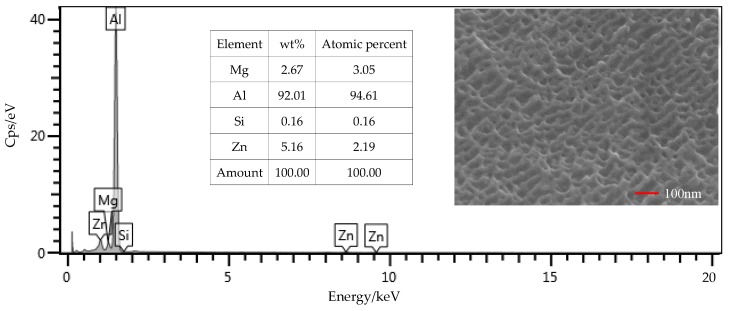
SEM and EDS test of the surface of the Al alloy sheet after the alkaline cleaning and acid pickling.

**Figure 10:**
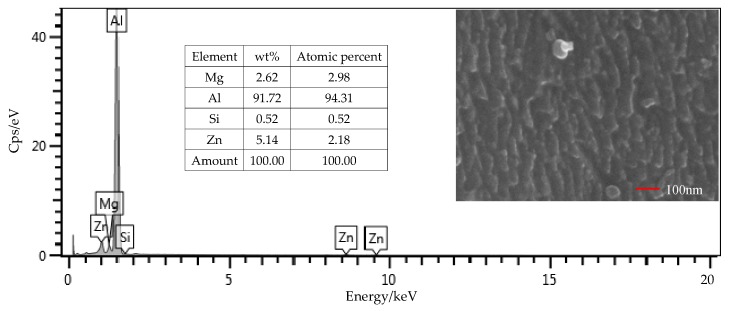
SEM and EDS test of the surface of the Al alloy sheet after the silanization treatment.

From the EDS results shown in Figure 9, the contents of the main components, including Mg, Si, and Zn, were all within the ranges presented in Table 1. The mass fraction of Al was 92.01%, which was slightly larger than the value of 91.4% in Table 1. Considering contents of the other elements that were not presented in the figure, this deviation was under a permissible level. Therefore, the Al alloy used in this study was in a qualifying condition. It is noted that the Si content was 0.16%, which was the initial content of the Al alloy sheet. From Figure 10, that content was increased to 0.52% on the surface of the Al alloy after the silanization treatment. From the structural formula in Figure 2, the Si element was present in the silane coupling agent KH560, and the Si atom was the central atom of the molecule. In the silanization process, the Si atom formed the Si–O–Al bond with the surface of the Al alloy by condensation reaction, and thus the silane coupling agent was grafted. An increase of the Si content confirms that the silane coupling agent was successfully grafted on the surface of the 7075 Al alloy sheet [17]. The results illustrate that the surface of the Al alloy sheet was successfully modified by the silane coupling agent, which served as a bridge for joining the organic adhesive to the inorganic Al alloy. As a result, the shear strength of the joints in Group 2 was increased significantly, from 6.43 MPa to 20.29 MPa.

The surface morphology of the Al alloy sheet after the alkaline cleaning and acid pickling is shown in Figure 9. From the figure, small pits with the length of about 100 nm were etched on the surface. After the silanization treatment, the surface was much rougher, and grooves with the length of about 200 nm were produced, as shown in Figure 10. It can be seen that the surface was further etched in the silanization treatment. This can be attributed to the silane coupling agent solution, which was acidic. The solution was a mixture of methanol, distilled water, and KH560 with the volume ratio of 96:3:1, and its pH value was adjusted to 5 with acetic acid. The acidic solution was beneficial to the hydrolyzing of the silane coupling agent. The Al alloy sheet was immersed in the silane coupling agent solution for 30 min at room temperature, and then kept in a vacuum oven at 110 °C for 1 h to finish the coupling reaction. Because of the long-time immersion of the Al alloy sheet in the acidic solution, the surface was etched. In addition, the condensation reaction on the surface also affected the grooves. In a certain range, the rougher morphology can promote mechanical anchoring at the interface between Al alloy and the adhesive, thus improving the shear strength.

### 3.3. Reaction at Interface

**Figure 11:**
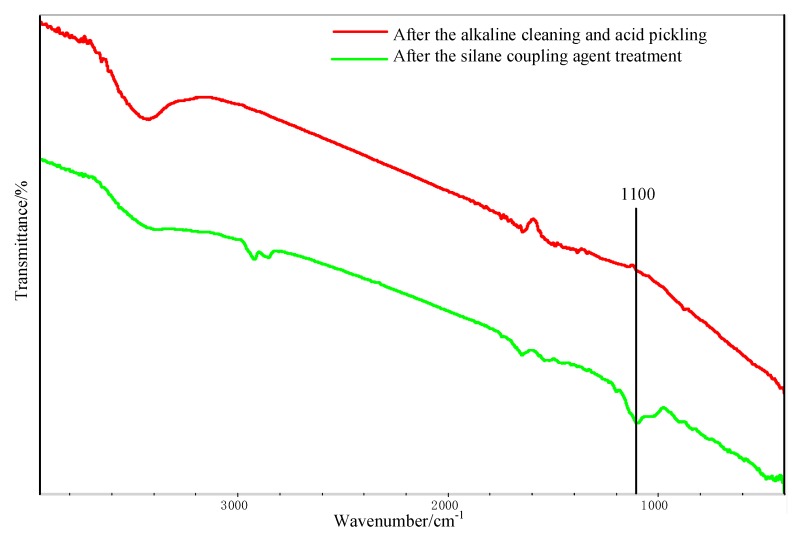
Fourier transform infrared spectrometer (FTIR) spectra of the Al alloy surface.

**Figure 12:**
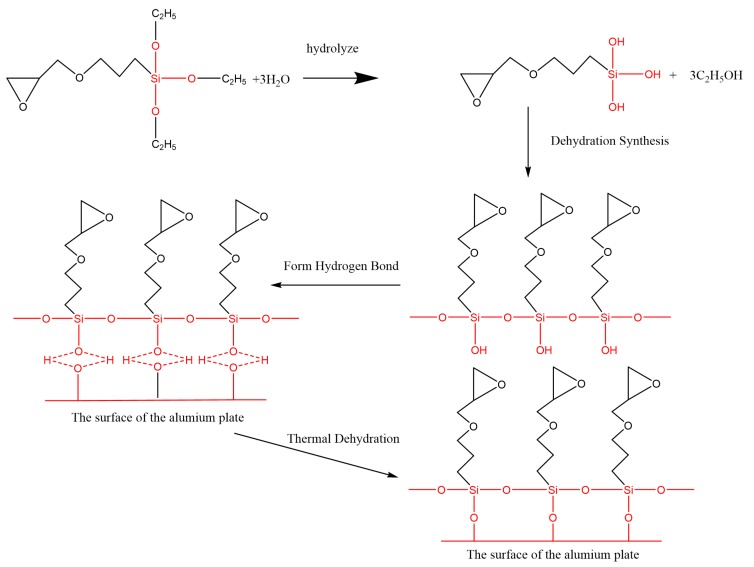
Condensation reaction of the silane coupling agent on the surface of the Al alloy sheet.

Figure 11 shows the FTIR test result of the Al alloy surface after the treatment of the alkaline cleaning plus acid pickling. The characteristic peak of hydroxyl was detected at 3423 cm^−1^, indicating that the hydroxyl group was formed on the surface of Al alloy after the treatment. After the silanization treatment, a peak at 1100 cm^−1^ was detected, as shown in Figure 11. This peak is the characteristic peak of the Si–O–Al bond. At the same time, the peak of hydroxyl is very weak, indicating that the hydroxyl group was consumed in the silanization treatment. In the treatment, the condensation reaction was carried out between the hydroxyl group on the grafted surface and that from the hydrolyzed silane coupling agent to produce the Si–O–Al bond, thus forming a chemical bridge between the inorganic Al alloy and the organic adhesive. The grafting reaction is shown in Figure 12. From the figure, it is noticed that the terminal epoxy group was also introduced on the surface of Al alloy after the treatment. From Figure 11, a peak was detected at 915 cm^−1^, which corresponds to the characteristic peak of the terminal epoxy group. Similar with the epoxy group in the adhesive, the grafted epoxy group can also react with the adhesive to form a chemical bond between the grafted surface and the adhesive layer, increasing the bonding strength.

**Figure 13:**
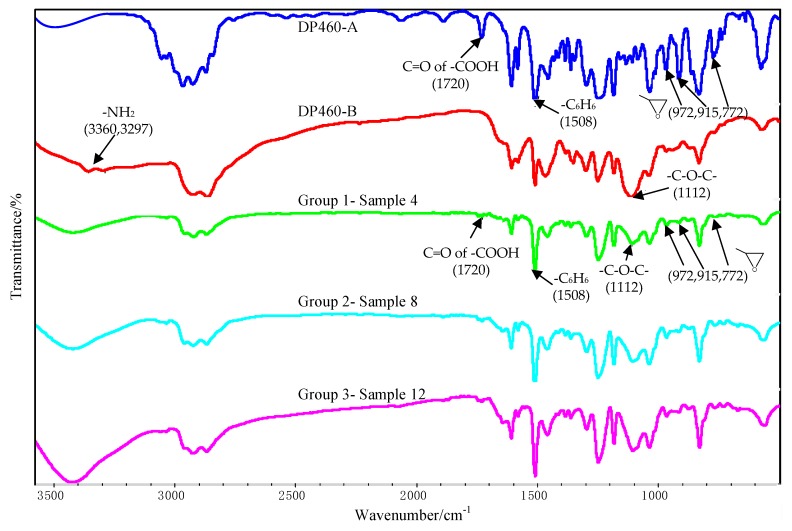
FTIR spectra of DP460-A/DP460-B and the interface between the Al alloy sheet and the adhesive.

The FTIR test results of the component A and the component B of the DP460 adhesive are shown in Figure 13. The peaks at 972 cm^−1^, 915 cm^−1^, and 772 cm^−1^ arose from stretching vibration of the terminal epoxy group. The peak at 1508 cm^−1^ arose from bending vibration of the para-substituted phenyl group. The peak at 1112 cm^−1^ was attributed to vibration of the ether group –C–O–C–. The peaks at 3360 cm^−1^ and 3297 cm^−1^ arose from vibration of the primary amine. Additionally, the absorption peak of C=O in the carboxylic acid was at 1720 cm^−1^. These absorption peaks confirm that the adhesive originally contains functional groups of terminal epoxy, phenyl, ether, primary amine, and carboxylic acid.

The FTIR test of the interface was conducted for the three bonded samples, and the spectra are shown in Figure 13, where Sample 4, Sample 8, and Sample 12 belong to Group 1, Group 2, and Group 3, respectively. The characteristic peaks of the epoxy group almost disappeared at 972 cm^−1^, 915 cm^−1^, and 772 cm^−1^, indicating that the epoxy was consumed during the cross-linking reaction with functional groups in the component B. From Figure 13, the peaks of the primary amine at 3297 cm^−1^ and 3360 cm^−1^ disappeared, indicating that the diethylene glycol bis (3-aminopropyl) ether in the adhesive was consumed during the cross-linking reaction.

**Figure 14:**
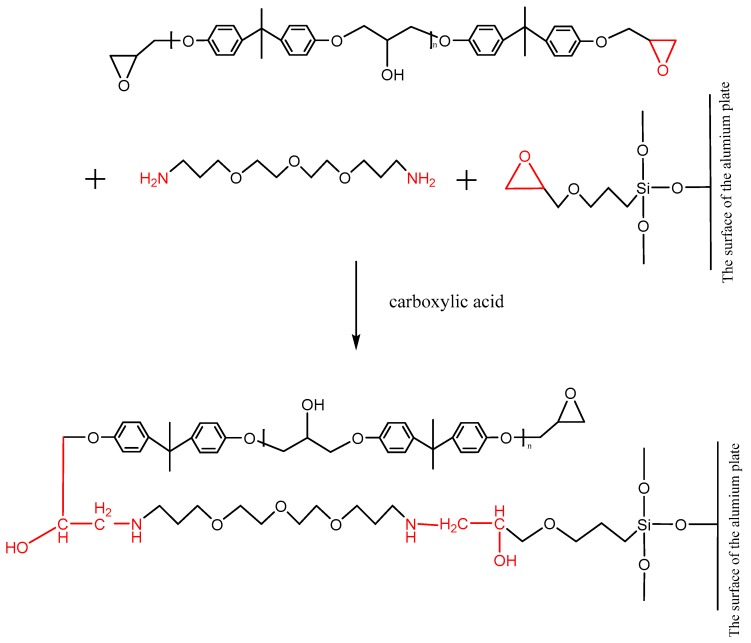
Reaction of the primary amine with the epoxy group on the surface of the grafted Al alloy.

The ether is an aliphatic amine hardener, containing the primary amine group, which can promote a ring opening reaction of the epoxy group at room temperature. The adhesive, whose main content is the bisphenol A epoxy resin that contains the epoxy group at both terminals of its molecule, can be cross-linked under the effect of the ether. Furthermore, after the silanization treatment, the surface of the Al alloy sheet was grafted with the silane coupling agent KH560, which contained the epoxy group at the terminal of its molecule, as shown in Figure 14. The ether promoted the ring opening reaction of the epoxy group to form a chemical bond between the grafted surface and the adhesive layer. The carboxylic acid, as an accelerator, can accelerate the reaction rate. The reaction formula is shown in Figure 14.

**Figure 15:**
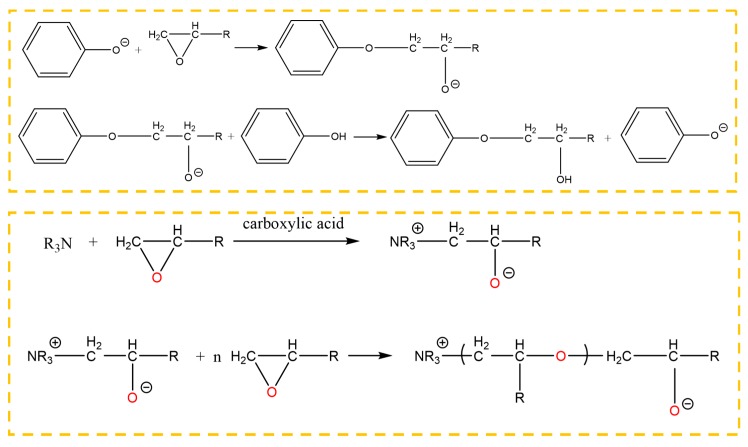
Reaction between 2,4,6-tris (dimethylaminomethyl) phenol and the epoxy group.

In the absence of a catalyst, a chemical reaction does not occur between phenol and epoxide until the temperature rises up to 200 °C. There were two kinds of reactions, that is, the reaction between the phenolic hydroxyl group and the epoxy group, and the reaction between the hydroxyl group generated in the ring opening reaction and the epoxy group. The first reaction was mainly carried out under a catalyst. The 2,4,6-tris (dimethylaminomethyl) phenol is an aromatic amine, in which the tertiary amine and the phenolic hydroxyl group are contained. The tertiary amine group, which is a Lewis base, acted as a catalyst to promote the reaction between the phenol and the epoxide at a low temperature. Owing to the tertiary amine group, the phenolic hydroxyl group lost a proton, promoting ring opening of the epoxy group. In addition, the anionic homopolymerization of the epoxy group was also carried out under the effect of the tertiary amine and the carboxylic acid. The reactions between the phenol and the epoxy resin are shown in Figure 15, where the epoxy group was from the grafted surface and the adhesive. Therefore, the chemical bond was formed at the interface.

After the silanization treatment, the surface of the Al alloy sheet was grafted with the silane coupling agent KH560, which contained the epoxy group at the terminal of its molecule. From Figure 14 and Figure 15, the grafted epoxy group reacted with the diethylene glycol bis (3-aminopropyl) ether and the 2,4,6-tris (dimethylaminomethyl) phenol in the adhesive to form a chemical bond between the Al alloy and the adhesive layer, thus improving the strength of the joints prepared using the silanization treatment.

The OMNIC software was employed to analyze Figure 13, and the peak area tool was used to obtain the peak area. Because the phenyl group was not involved in the chemical reactions, the area ratio of the epoxy peak to the phenyl peak was used to characterize degree of the cross-linking reaction. The characteristic peak of the terminal epoxy group is at 915 cm^−1^, and that of the phenyl group is at 1508 cm^−1^. The result is shown in Table 4. The ratio for Sample 4 was the smallest, indicating that the residual epoxy group was very little after the cross-linking reaction for the used adhesive. More residual epoxy group was measured in the other two samples according to the larger ratios. This was attributed to the silanization treatment applied for the two samples. The coupling agent of KH560 contains the terminal epoxy group, as shown in Figure 2. Extra epoxy group was grafted on the surface of the Al alloy after the silanization treatment, so that the residual epoxy group was more in Sample 8 and Sample 12. However, the residual epoxy group was less in Sample 12 than that in Sample 8, for the ratio was reduced from 4.17% to 3.60%, indicating that more grafted epoxy group was involved and consumed in the reaction with the adhesive. Considering that the additionally introduced epoxy group was very little, the reduction of the residual epoxy group was significant for the grafted epoxy group on the surface of the Al alloy. By comparing the surface treatment and joining process applied for the two samples, it is known that the ultrasonic vibration assisting process promoted the grafted epoxy group to react with the adhesive more sufficiently at the Al/adhesive interface. More chemical bond was formed between the grafted surface and the adhesive layer under the action of the ultrasonic vibration, thus further improving the strength of the CFRP/Al alloy joints.

From the view of the physical chemistry, the reason for the increase of the shear strength by the assisting process was that the ultrasonic action further promoted the reaction on the grafted surface to be finished completely. From Figure 13, the peaks were similar for the three bonded samples, indicating that the ultrasonic action did not change the types of chemical reactions at the interface. The ultrasonic action caused oscillation in the adhesive, inducing micro-mixing of materials [28]. Under the effect, the components of the adhesive could be mixed adequately, creating enough opportunity for the functional groups to collide and combine with each other, which increased the reaction probability of the grafted epoxy group at the interface. Owing to the mass transfer, more amine group (of the ether) was present at the interface. The group can promote ring opening reaction of the epoxy group readily. Therefore, more grafted epoxy group reacted to form a chemical bond between the adherend surface and the adhesive layer. Furthermore, under the ultrasonic action, high frequent vibration of the adherend and the adhesive was induced at the interface, so a high frequent impact between them was produced [26]. In such a condition, the probability that the phenolic hydroxyl group and the tertiary amine group (of the phenol) of the adhesive attacked the electrophilic C– on the grafted epoxy group was increased significantly. According to the curing mechanism of the bisphenol A epoxy resin, it can be concluded that the intense attack of the phenolic hydroxyl group and the tertiary amine group to the C on the epoxy promoted the ring opening reaction to form more chemical bond between the grafted surface and the adhesive. Under the effect of the ultrasonic action, the collision among the functional groups was intensified, also known as the thermal effect [29], which decreased the activation energy of the reactions at the interface. Such conditions facilitated some chemical reactions that were normally slow at the interface. Therefore, the reaction between the epoxy group and the adhesive was promoted at the grafted surface by the ultrasonic action.

### 3.4. Interface Morphology 

**Figure 16:**
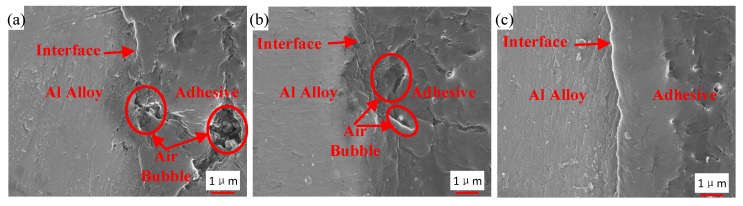
Interface morphology of the samples from (**a**) Group 1, (**b**) Group 2, and (**c**) Group 3.

Figure 16 shows the interface morphology of the bonded samples prepared by the three different processes. Air traps are observed in the adhesive layer and at the interface for the samples that were not strengthened by the ultrasonic vibration assisting process, as shown in Figure 16a,b. By comparing Figure 16a,b, air traps were decreased at the interface owing to the grafted surface of the sample in Figure 16b, but those were not improved dramatically in the adhesive layer. The area of max hollow is 6 μm^2^ in Figure 16a, and 5 μm^2^ in Figure 16b. Under the action of the ultrasonic vibration, no obvious hollow was observed, as shown in Figure 16c. The interface and the adhesive layer become tighter than those without the ultrasonic action. The vibration of the adhesive, caused by the ultrasonic action, produced impact contact at the interface between the adherend and the adhesive [26]. Ultrasonic vibration made it easier for the adhesive to permeate into microstructure of adherend surfaces, because the permeation was driven by the hydraulic pressure difference produced by the prompted flow of the adhesive [27]. In addition, under the effect of the ultrasonic vibration, air traps in the adhesive layer were decreased. The high-frequency vibration could induce oscillating flow in the adhesive layer. Because of internal pressure of air traps and asymmetric characteristic of fluid resistance around, the oscillating flow caused entrapped bubbles to break, move, and escape from the viscous adhesive [30], and thus air traps were decreased. Owing to the tight interface and adhesive layer, the interface adhesion of the adhesive and the Al adherend is more reliable.

**Figure 17:**
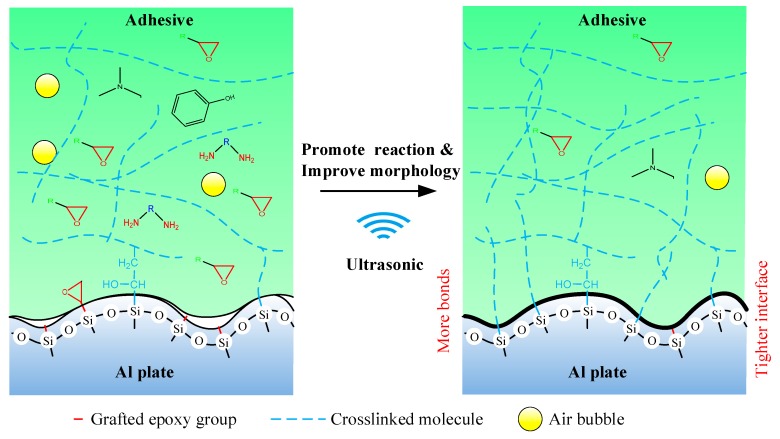
Mechanism of the ultrasonic assisting.

Finally, the mechanism of the ultrasonic vibration assisting process can be illustrated in Figure 17. The ultrasonic vibration assisting increased the shear strength by promoting the chemical bond and improving physical morphology. The ultrasonic assisting promoted the grafted epoxy group to react with the adhesive more sufficiently at the Al/adhesive interface, and thus more chemical bond was formed. Under the ultrasonic action, the interface and the adhesive layer became tighter.

## 4. Conclusions

Ultrasonic vibration was used to improve the adhesive bonding of CFRP/Al alloy joints grafted with silane coupling agent. The effect of the ultrasound on the bonding was studied. By comparing the strength, characteristic functional groups, and microstructure of the joints prepared by different surface treatments and joining processes, the following conclusions were drawn:

1. The ultrasonic vibration assisting can further promote the bonding of the Al alloy and the adhesive. For the employed joints, the shear strength was increased by 215.55% using the silanization treatment, but it was increased by 267.50% using the silanization treatment plus the ultrasonic vibration assisting. The damage of the ultrasonic strengthened joints exhibited the light-fiber-tear failure at the adhesive/CFRP adherend interface rather than the adhesive failure at the Al/adhesive interface.

2. The ultrasonic vibration assisting process promoted the grafted epoxy group to react with the adhesive more sufficiently at the Al/adhesive interface by causing the micro-mixing and intensified molecule collision, and thus more chemical bond was formed between the grafted surface and the adhesive layer, which improved the strength of the CFRP/Al alloy joints.

3. Under the ultrasonic action, the interface and adhesive layer became tighter, resulting in the more reliable interface adhesion. The vibration of the adhesive produced impact contact at the interface, resulting in the tight interface. The oscillating flow in the adhesive layer caused entrapped bubbles to break, move, and escape from the viscous adhesive, and thus air traps were decreased.

The ultrasonic vibration assisting process increases the bonding strength by promoting the chemical bond and improving physical morphology, which is of guiding significance for continuous optimization of the adhesive bonding.

## Figures and Tables

**Table 1:** Chemical composition of the 7075 Al alloy.

Element	Al	Zn	Mg	Cu	Fe	Si	Cr	Others
Mass fraction/%	87.1–91.4	5.1–6.1	2.1–2.9	1.2–2.0	≤0.5	≤0.4	0.18–0.28	≤0.65

**Table 2:** Three experiments of different surface treatments and joining processes.

	Sample Label	Surface Treatment of Al Alloy	Joining Process
Group 1	Sample 1, 2, 3, 4	Sanding + Alkaline cleaning + Acid pickling + Ultrasonic cleaning + Drying	Adhesive bonding
Group 2	Sample 5, 6, 7, 8	The surface treatment illustrated in Figure 5	Adhesive bonding
Group 3	Sample 9, 10, 11, 12	The surface treatment illustrated in Figure 5	Adhesive bonding + Ultrasonic vibration

**Table 3:** Shear strength of the samples for each group.

Group	Sample Number	Failure Load (N)	Shear Strength (MPa)	Mean Value (MPa)
1	1	3517.34	5.45	6.43
	2	4087.14	6.34	
	3	4841.39	7.50	
2	5	12,512.89	19.40	20.29
	6	14,690.39	22.77	
	7	12,070.91	18.71	
3	9	14,332.97	22.22	23.63
	10	16,423.38	25.46	
	11	14,984.90	23.23	

**Table 4:** Peak area of the two functional groups.

Group	Sample Number	Peak Area of Epoxy Group	Peak Area of Phenyl Group	Ratio
1	4	3.12	93.13	3.35%
2	8	6.47	154.61	4.17%
3	12	2.86	79.56	3.60%
